# Distinct Calcium Binding and Structural Properties of Two Centrin Isoforms from *Toxoplasma gondii*

**DOI:** 10.3390/biom10081142

**Published:** 2020-08-04

**Authors:** Luca Bombardi, Marco Pedretti, Carolina Conter, Paola Dominici, Alessandra Astegno

**Affiliations:** Department of Biotechnology, University of Verona, Strada Le Grazie 15, 37134 Verona, Italy; luca.bombardi@univr.it (L.B.); marco.pedretti@univr.it (M.P.); carolina.conter@univr.it (C.C.); paola.dominici@univr.it (P.D.)

**Keywords:** centrin, *Toxoplasma gondii*, EF-hand, calcium sensor, conformational change, self-assembly

## Abstract

Centrins are calcium (Ca^2+^)-binding proteins that have been implicated in several regulatory functions. In the protozoan parasite *Toxoplasma gondii*, the causative agent of toxoplasmosis, three isoforms of centrin have been identified. While increasing information is now available that links the function of centrins with defined parasite biological processes, knowledge is still limited on the metal-binding and structural properties of these proteins. Herein, using biophysical and structural approaches, we explored the Ca^2+^ binding abilities and the subsequent effects of Ca^2+^ on the structure of a conserved (TgCEN1) and a more divergent (TgCEN2) centrin isoform from *T. gondii*. Our data showed that TgCEN1 and TgCEN2 possess diverse molecular features, suggesting that they play nonredundant roles in parasite physiology. TgCEN1 binds two Ca^2+^ ions with high/medium affinity, while TgCEN2 binds one Ca^2+^ with low affinity. TgCEN1 undergoes significant Ca^2+^-dependent conformational changes that expose hydrophobic patches, supporting a role as a Ca^2+^ sensor in toxoplasma. In contrast, Ca^2+^ binding has a subtle influence on conformational features of TgCEN2 without resulting in hydrophobic exposure, suggesting a different Ca^2+^ relay mode for this isoform. Furthermore, TgCEN1 displays a Ca^2+^-dependent ability to self-assemble, while TgCEN2 did not. We discuss our findings in the context of Ca^2+^ signaling in toxoplasma.

## 1. Introduction

Centrins are calcium (Ca^2+^)-binding proteins that are ubiquitously expressed in eukaryotes and commonly associated with the microtubule-organizing center (MTOC), which is the centrosome in mammals, the spindle pole body in yeast, and the basal body in ciliated and flagellated cells. However, only a small percentage of total centrins (~10%) is restricted to MTOCs, and the remaining percentages are distributed more diffusely in other organelles and cellular compartments [1]. Their different intracellular localizations may be associated with the different functions that have been reported. Indeed, centrins are crucial proteins that are involved in many physiological pathways, such as DNA repair, mRNA export, centrosome duplication, and signal transduction [2,3,4,5,6,7]. Although in the yeast *Saccharomyces cerevisiae* a single centrin is responsible for such functional diversity, various centrin isoforms have been identified in other organisms, comprising higher plants and mammals [8,9,10,11], and biochemical and structural analyses have indicated distinct molecular properties for diverse centrin isoforms [4,6,12,13,14,15].

Similar to calmodulin (CaM), centrins generally fold into two independent domains, the N-lobe and the C-lobe, each of which harbors a pair of characteristic helix-loop-helix structures, called the EF-hands, which constitute highly specific potential Ca^2+^-binding sites. In the canonical EF-hand, a Ca^2+^ ion is coordinated by seven oxygen-containing groups, five provided by amino acids at positions 1, 3, 5, 7, and 9 in the loop and two by bidentate chelation of a glutamate at position 12 outside of the loop. At positions 1 and 3, aspartate or asparagine are usually found, while at position 5, aspartate, asparagine, or serine are most commonly present. Moreover, two non-coordinating residues are crucial in most EF hands; a glycine at position 6, which confers flexibility in the Ca^2+^ site and a hydrophobic residue, such as isoleucine, valine, or leucine at position 8. However, variability in residue composition within the EF-hand sequence may be present in centrins from various organisms, resulting in different coordination mechanisms, affinity, or specificity of the EF-hand for Ca^2+^. For example, the substitution of glutamate with an aspartic acid residue at position 12 is known to cause a decrease in Ca^2+^ affinity, shifting the binding selectivity from Ca^2+^ towards Mg^2+^ ions [16,17]. The selective binding of Ca^2+^ to the EF-hands of centrin usually causes a conformational change from an apo (closed) state to a holo (open) state causing solvent exposure of a hydrophobic region that is responsible for molecular recognition and interaction with cellular targets. A major difference between CaM and centrins is the presence of an extended unstructured 20–25 residues at the N-terminal domain of centrins that may have specialized a biological function in self-assembly and subcellular localization of the protein. 

In *Toxoplasma gondii*, a protozoan parasite of the phylum Apicomplexa that causes toxoplasmosis in all warm-blooded animals, including humans, three centrin isoforms have been identified, namely centrin 1 (TgCEN1), centrin 2 (TgCEN2), and centrin 3 (TgCEN3) [18,19,20], suggesting that the centrins in the parasite may have various biological functions. As with centrins in other organisms, TgCEN1 and TgCEN3 are mainly restricted to the centrioles. Though TgCEN2 possesses high sequence similarity with TgCEN1 (~50% sequence identity) and TgCEN3 (~46% sequence identity), it is also found in various cytoskeletal components at the apical and basal portions of the parasite, where it has been demonstrated to function in various aspects of parasite physiology, including secretion, invasion, and replication [19,21,22]. However, little is known about the structural properties of centrins from *T. gondii* and how they are affected by Ca^2+^. This could represent crucial information to better understand how these proteins participate in relevant cellular processes and whether they, as EF-hand containing proteins, are part of Ca^2+^ signaling pathways that control parasite physiology.

In the present study, using an array of complementary biophysical and biochemical approaches, we investigated the molecular properties of the recombinant proteins TgCEN1 and TgCEN2 with the specific aim to elucidate the role of the Ca^2+^ ion in determining the structural properties of the proteins and to understand the metal binding, thermodynamic, and structural differences between the two isoforms. TgCEN3 was not included in the analysis because its recombinant production resulted in an insoluble protein and, unfortunately, our efforts to solubilize it have been unsuccessful to date. 

The obtained results might have far-reaching consequences for the functional understanding of the mechanism of action of TgCEN1 and TgCEN2, paving the way for subsequent strategies of parasite disruption through targeting of centrins in treating toxoplasmosis.

## 2. Materials and Methods 

### 2.1. Protein Production

Synthetic genes (GenScript USA Inc., Piscataway, NJ, USA) corresponding to the complete cDNA of TgCEN1 (TGME49_247230) and TgCEN2 (TGME49_250340) with a tag of six His at the N-terminal were cloned into a pET21a expression vector using NdeI and BamHI as restriction sites. The recombinant vectors were transformed into DH5α-derived *E. coli* cells made competent by the CaCl_2_ method. TgCEN1 lacking the first 21 residues (residues 22–169, named TgCEN1-C) and TgCEN2 lacking the first 22 amino acids (residues 23–170, named TgCEN2-C) were generated by site specific mutagenesis on the pET21a-TgCEN1 and pET21a-TgCEN2 wild type constructs, respectively, using the QuikChange^®^ site-directed mutagenesis kit (Agilent Technologies, Santa Clara, CA, USA), according to the manufacturer’s recommendations for multiple-site deletion in a single step. The G43A, G79A and G152A TgCEN1-C and the G44A, G153A TgCEN2-C single-site variants were produced by site specific mutagenesis on the wild type constructs using the QuikChange^®^ site-directed mutagenesis kit. The primer sequences used are listed in Supplementary Table S1. The mutated sequences were confirmed by DNA sequence analysis.

The verified plasmids for full-length, N-terminal deletion, and site-specific variants of centrins were transformed into *E. coli* Rosetta (DE3) expression host cells. Each culture was grown at 37 °C to OD_600_ of 0.6 and induced with 0.5 mM IPTG (isopropyl-β-d-thiogalactoside) at 24 °C for 18 h. The cells were harvested by centrifugation (5000 *g* for 15 min at 4 °C) and resuspended in 50 mM Tris-HCl, 150 mM KCl, 10 mM imidazole, pH 7.5 in the presence of an EDTA-free protease inhibitor. After sonication and centrifugation, the supernatant of lysed cells was collected and applied to a Ni-chelating column equilibrated with 50 mM Tris-HCl, 150 mM KCl, 10 mM imidazole, pH 7.5. Protein elution was performed using a linear gradient from 10 to 500 mM imidazole. Purified centrins were extensively dialyzed in 50 mM Tris-HCl, 150 mM KCl, pH 7.5 buffer to remove imidazole. The homogeneity and purity of the protein variants were verified by SDS-PAGE and protein concentration was determined using the Bradford assay and/or biuret reaction [23,24]. 

The purification and all of the following experiments with TgCEN1 were performed by adding 0.5 mM DTT to buffers due to the presence of a cysteine residue in the protein.

^15^N labeled proteins were prepared in the same manner, except that they were expressed in M9 minimal medium enriched with 1 g/L ^15^NH_4_Cl as the sole source of nitrogen.

Purified recombinant proteins were assayed by native PAGE (12%) and SDS-PAGE (15%) after a 30-min reaction at 25 °C with 2 mM CaCl_2_ or 2 mM EGTA, as described [6,25].

### 2.2. Nuclear Magnetic Resonance Spectroscopy

Nuclear magnetic resonance (NMR) experiments were performed at 25 °C with approximately 0.4 mM protein samples in 50 mM Tris-HCl, 50 mM KCl, 10% D_2_O pH 7.5 on a Bruker Avance III spectrometer, operating at 600.13 MHz proton Larmor frequency and equipped with a cryogenic probe. ^1^H-^15^N HSQC (heteronuclear single quantum coherence) spectra were collected by a standard pulse sequence. The instrument data matrix consisted of 2 K (F2,1H) × 256 (F1,15N) complex points, spectral windows of 9615.38 Hz (1H) × 2432.71 Hz (15N), 4 or 8 transients, and 1.2 s relaxation delay. NMR data were processed with Topspin 3.6 (Bruker).

### 2.3. Circular Dichroism Spectroscopy

Purified proteins were analyzed by far-UV CD spectroscopy (wavelength range 200–250 nm) on a Jasco J-1500 spectropolarimeter equipped with temperature control of the sample compartment. The spectra were measured in 50 mM Tris-HCl, 150 mM KCl pH 7.5 in the presence of 2 mM CaCl_2_ or 2 mM EGTA, at a protein concentration of 0.2 mg/mL at 25 °C using a 0.1 cm quartz cuvette. An average of five accumulations were measured, using a scan rate of 50 nm/min. Spectra were corrected by subtraction of the buffer signal. Thermal unfolding profiles were obtained by monitoring the CD signal at 222 of apo- and Ca^2+^-bound proteins over a temperature range from 15 °C to 90 °C at a heating rate of 1.5 °C/min. Mean values ± standard error of the mean (SEM) of melting temperature were obtained from triplicate experiments.

### 2.4. Limited Proteolysis

Proteins (0.5 mg/mL) were treated with trypsin (1:500 w/w) in 50 mM Tris-HCl, 150 mM KCl pH 7.5 after the addition of 2 mM CaCl_2_, or 2 mM EGTA at 25 °C. At different time intervals (0, 1, 5, 10, 20, 60, 90, and 120 min), 10 μL aliquots of proteolysis mixtures were boiled (in the presence of reducing Laemmli buffer) to quench the reaction and analyzed by SDS-PAGE. 

### 2.5. Size Exclusion Chromatography

The apparent molecular mass of the purified apo- and Ca^2+^-bound proteins (2 mg/mL) in solution was determined by size exclusion chromatography using a Superdex 75 Increase 10/300 GL column (GE Healthcare Europe GmbH, Milano, Italy) in 50 mM Tris-HCl, 150 mM KCl pH 7.5. A calibration curve was constructed using the following standard proteins (GE Healthcare gel filtration calibration kit): cytochrome c (12 kDa); myoglobin (17 kDa); carbonic anhydrase (29 kDa); ovalbumin (43 kDa) and albumin bovine serum (66 kDa) as described [26,27]. The void volume was obtained using blue dextran (2000 kDa). The values reported are the mean ± SEM of triplicate experiments.

### 2.6. Fluorescence Spectroscopy

Fluorescence emission measurements using 1-anilino-8-naphthalenesulfonic acid (ANS) were conducted on a Jasco FP8200 spectrofluorometer as previously described [28]. A fixed concentration of 15 μM ANS was mixed with 1 μM protein solution in 50 mM Tris-HCl, 150 mM KCl pH 7.5, in the presence of 2 mM EGTA or 2 mM CaCl_2_. The emission spectra were collected from 400 nm to 650 nm after excitation at 380 nm. All measurements were performed in triplicate and the mean values ± SEM are given.

### 2.7. Isothermal Titration Calorimetry

Isothermal titration calorimetry (ITC) measurements were conducted on TA Instrument Nano ITC. A typical experiment was carried out on a protein solution (0.2 mL of apo-proteins at 70–100 µM) by adding 2 mM CaCl_2_ in 33 aliquots (1.5 µL each) at 300 s intervals at 25 °C. A first injection of 0.5 μL was made and then the first data point was removed from data fitting. Decalcified ITC buffer (50 mM Tris-HCl, 150 mM KCl pH 7.5) was prepared by processing with Chelex 100 resin (BioRad, Hercules, CA, USA) [29,30]. The sample cell and injection syringe were washed extensively with decalcified buffer. All solutions were degassed before each experiment. Injections of CaCl_2_ solution into the buffer without any protein (buffer blank titration) was performed but did not show significant heat-related changes in the recording cell. Data was analyzed using Origin-based software. One-site and two-site binding site models were fitted to the data. Of these attempted binding models, it was found that the two-site model provided the best fit for TgCEN1-C data while for TgCEN2-C the best fit was obtained using the one-site model, as determined by the reduced chi squared value. Mean values ± SEM of at least three independent titrations on different protein preparations were reported.

### 2.8. Turbidity Measurements

The self-assembly propensity of centrins (20 μM) was monitored by turbidimetry experiments measuring the change in the absorbance at 340 nm as a function of time at 37 °C. All spectroscopic measurements were performed using a Jasco V-560 spectrophotometer with 1-cm path length quartz cuvettes in 20 mM Tris-HCl pH 7.5 and different KCl concentrations. 

### 2.9. Dynamic Light Scattering

Dynamic light scattering (DLS) measurements were performed on a Zetasizer Nano S instrument (Malvern Instruments, Malvern, UK) equipped with a Peltier temperature controller by using disposable 12.5 × 45 mm cells with a stopper. The self-assembly kinetics of centrins (10 μM) were determined at 37 °C before and after addition of 1 mM CaCl_2_ in 20 mM Tris-HCl pH 7.5 and different KCl concentrations. All of the buffers were filtered immediately before each measurement in order to eliminate any impurities. The increase in the mean count rate, a parameter that represents the average scattering intensity during the measurement and directly reports on aggregated species present in solution, was followed.

## 3. Results

### 3.1. Sequence Analysis and Protein Production

**Figure 1:**
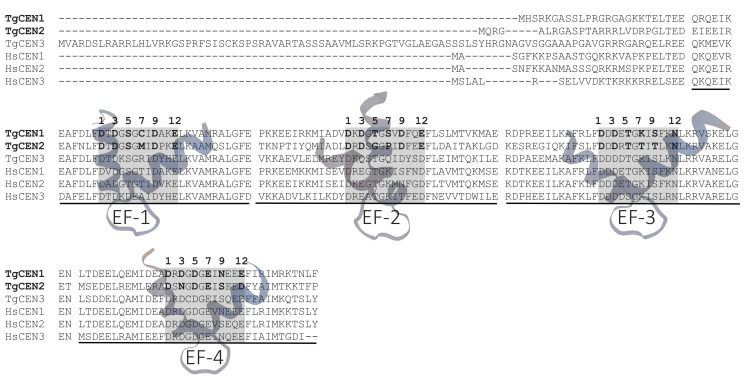
Multiple sequence alignments of TgCEN1 and TgCEN2 with other members of the centrin family. Residues critical for Ca^2+^ coordination (positions 1, 3, 5, 7, 9, and 12) in EF hands are shown in bold. TgCEN1 (*T. gondii* CEN1, UniProt: A0A125YHX7), TgCEN2 (*T. gondii* CEN2, UniProt: A0A125YZN2), TgCEN3 (*T. gondii* CEN3, UniProt: A0A125YXG1), HsCEN1 (*H. sapiens* CEN1, UniProt: Q12798), HsCEN2 (*H. sapiens* CEN2, UniProt: P41208), HsCEN3 (*H. sapiens* CEN3, UniProt: O15182). Sequences were aligned using Clustal Omega [34].

TgCEN1 and TgCEN2 are 169- and 170-amino-acid proteins with a predicted molecular mass of 19,343 Da and 19,153 Da, respectively, which is in the range of known centrins. Figure 1 shows the alignment of TgCEN1 and TgCEN2 with TgCEN3 and the centrin homologs in *H. sapiens* (HsCEN). The level of homology of TgCEN1 to the other centrin sequences revealed ~50% identity to TgCEN2, ~60% identity to TgCEN3, and ~70% identity to human centrins HsCEN1 and HsCEN2. TgCEN2 displayed a lower level of conservation, reaching only ~46% identity to TgCEN3 and ~50% identity to HsCEN1 and HsCEN2 (Figure 1).

TgCEN1 and TgCEN2 consist of eight and nine α-helices, respectively, as predicted by Jpred 4 online [31] and both possess four EF-hand motifs. However, analysis of amino-acid residues in the EF-hand loop region, by using the ScanProsite tool to find the pattern for Ca^2+^ binding domains, indicates that TgCEN1 is predicted to contain three (EF-1, EF-2 and EF-4) profiles of potential Ca^2+^-binding sites, while TgCEN2 is predicted to contain only two (EF-1 and EF-4) potential Ca^2+^-binding sites [32] (Figure 1). In both TgCEN1 and TgCEN2, the amino acid sequence of EF-3 does not fit the EF-hand consensus sequence because an asparagine is found in position 12 where a negative glutamate normally provides two oxygens to coordinate Ca^2+^. Moreover, in TgCEN2 the EF-2 lacks crucial residues required for Ca^2+^ binding. A glycine at position 5 of the loop in EF-2 cannot chelate Ca^2+^, which is thus expected to preclude physiological Ca^2+^ binding to EF-2. In addition, the presence of a proline at position 7 could prevent the stabilization of negative charges in the Ca^2+^ coordination sphere. Of note, the replacement of the conserved glutamate residue with an aspartate at position 12 in EF-4 of TgCEN2 can result in a dramatic drop in Ca^2+^ affinity, as demonstrated for other Ca^2+^ binding proteins [16,17,33], thus raising the possibility that TgCEN2 has only one predicted Ca^2+^ binding site.

Sequence diversity among centrins is prominently present in the N-terminal domain within the first 20–25 residues (before the beginning of the first EF-hand). In particular, this N-terminal string (residues 1–21 in TgCEN1 and 1–22 in TgCEN2, respectively) was analyzed by using GeneSilico MetaDisorder Service [35] and resulted in high intrinsic disorder. Notably, in other centrins, such as HsCEN2 [36], the N-terminal portion was found to be responsible for Ca^2+^-dependent self-assembly. 

Decreasing the molecular disorder, by removing the first 21 residues in TgCEN1 and the first 22 residues in TgCEN2, respectively, enabled us to significantly improve the quality of the data, especially for analyses requiring high protein concentrations (e.g., NMR spectroscopy). Thus, all the qualitative and quantitative analyses of the present study were performed using the deleted recombinant proteins (residues 22–169, named TgCEN1-C and residues 23–170, named TgCEN2-C). TgCEN1-C and TgCEN2-C were observed as single bands in SDS-PAGE (Figure S1A) corresponding to their molecular weight, suggesting a high level of purity. Notably, the mobility of both TgCEN1-C and TgCEN2-C slightly increased in the presence of Ca^2+^, which is a hallmark of Ca^2+^ binding proteins. A shift in mobility (more evident in the case of TgCEN1-C) was also seen under native conditions for both proteins, even if in the opposite direction (Figure S1B), as reported in the literature [37,38,39]. Importantly, under non-denaturing conditions, both apo- and Ca^2+^-bound TgCEN1-C and TgCEN2-C ran as single bands, indicating no formation of higher-order oligomers. For the sake of completeness, SDS-PAGE and native PAGE analysis of full-length TgCEN1 and TgCEN2 are also reported in Figure S1.

Since TgCEN1-C and TgCEN2-C were predicted to be Ca^2+^ binding proteins on the basis of EF-hand motifs and Ca^2+^-dependent electrophoretic mobility shift, we carried out a set of experiments to show whether these proteins could actually bind Ca^2+^, what effects the metal ion had on their structure, and to identify structural and thermodynamic differences between the two isoforms. 

### 3.2. NMR Spectroscopy

**Figure 2:**
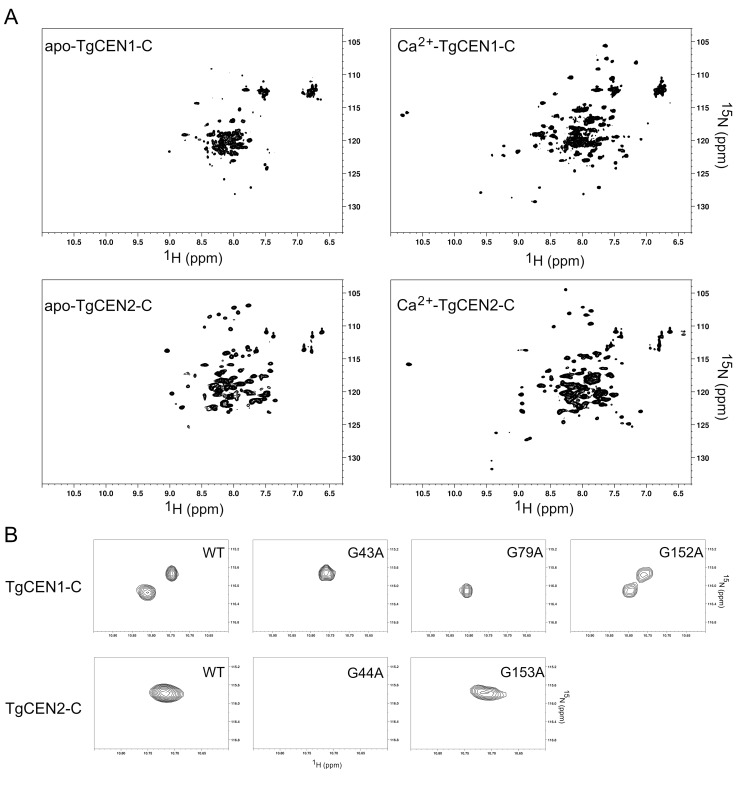
^1^H-^15^N HSQC NMR spectra of TgCEN1-C and TgCEN2-C. (**A**) NMR spectra of apo- or Ca^2+^-bound ^15^N-labelled TgCEN1-C or TgCEN2-C. The apo-condition was obtained by adding 5 mM EGTA, while the Ca^2+^-bound condition was obtained by adding 5 mM CaCl_2_. (**B**) Downfield spectral region of ^1^H-^15^N HSQC spectra showing Gly-6 peaks of TgCEN1-C and TgCEN2-C variants in the presence of 5 mM CaCl_2_.

Ca^2+^ binding to TgCEN1-C and TgCEN2-C was monitored by heteronuclear two-dimensional ^1^H-^15^N HSQC NMR spectroscopy (Figure 2A). The spectrum of apo-TgCEN1-C exhibited poor peak dispersion with few well resolved resonances. Addition of Ca^2+^ to the protein sample improved the chemical shift dispersion, expanding the number of visible signals and causing changes in the positioning of several resonances with peaks that were of more uniform intensity throughout the spectrum than those of apo-TgCEN1-C. These spectral changes suggest that the protein undergoes a large Ca^2+^-induced conformational change, likely adopting a somewhat more stable tertiary structure in the presence of Ca^2+^. It is interesting to note that several other centrins [6,14,40], as well as several related Ca^2+^-binding proteins [28,41,42,43,44], have strikingly similar HSQC NMR spectra with a greater dispersion of cross peaks for the Ca^2+^-loaded state than for the apo-state and with Ca^2+^ binding that provides the protein with important structural stability. A similar behavior was also observed for TgCEN2-C, even if upon addition of Ca^2+^ the protein appeared to undergo switching with minor changes in conformation compared to TgCEN1-C. Notably, the spectrum of apo-TgCEN2-C displayed an overall better chemical shift dispersion of resonances compared to the spectrum of apo-TgCEN1-C, suggesting that apo-TgCEN2-C is organized in a more ordered structure than apo-TgCEN1-C, consistent with the far-UV CD data (see Section 3.3). 

Two new signals in the spectrum of holo-TgCEN1-C and one new peak in the spectrum of holo-TgCEN2-C were clearly detectable in the downfield shifted ^1^H region, at 10.74 and 10.81 ppm for TgCEN1-C, and 10.71 ppm for TgCEN2-C, respectively. The signals in the low field-shifted ^1^H region generally arise from the invariant glycine residue at the sixth position (Gly-6) of the EF-hand Ca^2+^-binding loop, as a result of Ca^2+^ binding [28,41,43,44,45,46]. Thus, they usually constitute distinctive signs to recognize Ca^2+^-binding sites and to monitor protein folding. The emergence of two downfield shifted signals in holo-TgCEN1-C and one downfield shifted peak in holo-TgCEN2-C spectra recorded under in vitro conditions suggested the presence of at least two and one functional Ca^2+^-binding sites in TgCEN1-C and TgCEN2-C, respectively. Similar conclusions were reached by analyzing the NMR spectra of full-length TgCEN2 in the apo- and holo-states even if, as stated above, the spectra generally suffered from low resolution and low quality (Figure S2). On the other hand, addition of Ca^2+^ (even at low concentrations) to full-length TgCEN1 caused spectral overlap and a progressive loss of protein signal and resolution (data not shown), indicating that the protein may undergo strong aggregation and/or self-assembly in the presence of the ion (see Section 3.7). 

We also investigated the ability of the two isoforms to bind Mg^2+^, since different studies have shown that Mg^2+^ can bind to the Ca^2+^-binding sites of many centrins [13,47]. Importantly, the addition of Mg^2+^ to apo-TgCEN1-C and apo-TgCEN2-C did not substantially affect the HSQC spectra, which were similar to those in the presence of EGTA, implying that Mg^2+^ does not likely bind to either of the two proteins (Figure S3).

In order to assign a specific downfield shifted peak to the Gly-6 of a particular EF-hand, and thus identify the functional Ca^2+^ binding sites out of the four EF-hands in the two centrin isoforms, we performed site-specific mutagenesis of Gly-6 residues belonging to EF-1 (G43A), EF-2 (G79A) and EF-4 (G152A) in TgCEN1-C, and EF-1 (G44A) and EF-4 (G153A) in TgCEN2-C. By comparing ^1^H-^15^N HSQC spectra of wild type proteins to those of protein variants, we found that the two downfield shifted peaks in the spectrum of holo-TgCEN1-C correspond to G43 and G79, while the one in the spectrum of holo-TgCEN2-C belongs to the G44 residue (Figure 2B), demonstrating that Ca^2+^ is minimally bound at EF-1 and EF-2 in TgCEN1-C and at EF-1 in TgCEN2-C. Indeed, the NMR spectrum of the TgCEN1-C G43A mutant recorded in the presence of Ca^2+^ lacked the peak at 10.81 ppm seen in the wild type, while that of the TgCEN1-C G79A mutant lacked the peak at 10.74 ppm. Correspondingly, the ^1^H-^15^N HSQC spectrum of the TgCEN1-C G152A mutant did not show differences in the downfield region compared to wild type holo-TgCEN1-C. Analogously, in the HSQC spectrum of the ^15^N labelled TgCEN2-C G44A mutant, the signal at 10.71 ppm disappeared, while the NMR spectrum of the TgCEN2-C G153A mutant in the low field-shifted ^1^H region overlapped that of the wild type protein.

### 3.3. Far-UV CD Spectroscopy

**Figure 3:**
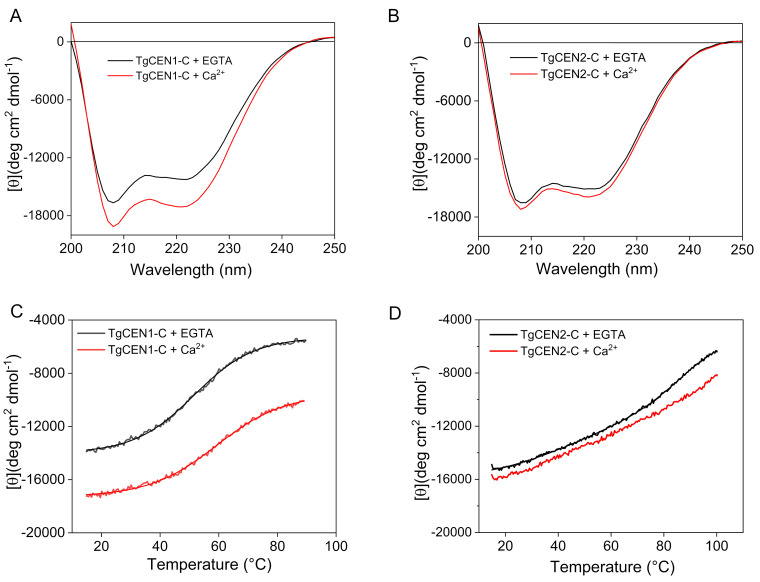
Far-UV CD spectra and thermal stability. (**A**,**B**) Far-UV CD spectra of 0.2 mg/mL TgCEN1-C (**A**) and TgCEN2-C (**B**) in the presence of 2 mM EGTA (black) and 2 mM CaCl_2_ (red). (**C**,**D**) Representative thermal denaturation profiles of TgCEN1-C (**C**) and TgCEN2-C (**D**) in the presence of 2 mM EGTA (black) and 2 mM CaCl_2_ (red).

The Ca^2+^-mediated conformational changes in the two centrin isoforms were further investigated by probing the secondary structure using far-UV (200–250 nm) CD spectroscopy. The CD spectra of TgCEN1-C and TgCEN2-C displayed two negative peaks with local minima around 208–209 and 221–222 nm typical of proteins containing a mainly α-helical structure (Figure 3A,B). Following Ca^2+^ binding, TgCEN1-C showed a significant increase in the elliptic signal, accompanied by a variation in the θ_222_/θ_208_ ratio, which switches from 0.85 in the apo-form to 0.90 in the holo-form (Figure 3A), which has been interpreted in the literature as reorientation/distortion of the existing α-helices in the tertiary structure and not simply to an increase in α-helical content [48]. The reduced θ_222_/θ_208_ value observed in apo-TgCEN1-C compared to that in the presence of Ca^2+^ supports the presence of some random and flexible structures in the apo-state of the protein, as deduced by NMR spectra On the other hand, upon Ca^2+^ binding, TgCEN2-C showed only small changes of the spectrum with a slight increase in molar ellipticity and no variations in shape, as illustrated by the θ_222_/θ_208_ ratio (0.91 for both apo- and Ca^2+^-bound TgCEN2-C) (Figure 3B). In both the apo- and holo-form, TgCEN2-C is clearly well-folded in solution.

Consistent with NMR results, binding of Mg^2+^ to both apo-centrins induced no significant changes in the far UV CD spectra (Figure S4).

Additionally, far-UV CD was used to investigate the thermal stability of TgCEN1-C and TgCEN2-C by following the CD signal at 222 nm (Figure 3C,D). Apo-TgCEN2-C was found to be very stable with a transition temperature (T_m_ = 84 ± 2 °C) that was ~30 °C higher than apo-TgCEN1-C (T_m_ = 51 ± 1 °C). Addition of Ca^2+^ increased the thermal stability for both proteins even if to a different extent. Indeed, holo-TgCEN1-C displayed a T_m_ = 60 ± 1 °C, while holo-TgCEN2-C did not reach a plateau, thus it was not possible to determine the inflexion point for the estimation of the T_m_. Notably, the presence of a CD signal even at 90 °C independent of the presence of EGTA or Ca^2+^ suggests that the two centrins preserve a partially folded structure even at this high temperature.

### 3.4. Limited Proteolysis and Size Exclusion Chromatography

As further ways to investigate conformational changes occurring upon Ca^2+^ binding, we performed limited proteolysis and size exclusion chromatography (SEC) experiments of TgCEN1-C and TgCEN2-C in the presence and absence of Ca^2+^. 

**Figure 4:**
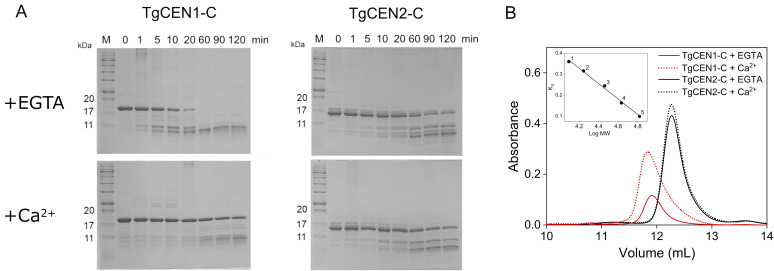
Representative tryptic digestions (**A**) and SEC profiles (**B**) of TgCEN1-C and TgCEN2-C in the presence of EGTA or CaCl_2_. (**A**) Tryptic digestion: the intensity of the untreated centrin band in lane 2 was assumed to be 100%. Lane 1: MW marker, lanes 2–9: trypsin digestion products obtained following incubation of protein with trypsin 1:500 w/w for 0, 1, 5, 10, 20, 60, 90 and 120 min, respectively. (**B**) SEC profiles: proteins were analyzed at 2 mg/mL on a Superdex 75 Increase 10/300 GL gel filtration column in buffer containing EGTA (5 mM; solid trace) or CaCl_2_ (5 mM; dotted trace). *Inset*, calibration curve of distribution coefficient K*d* versus the logarithm of the molecular weight. The standard proteins used were: 1, cytochrome c; 2, myoglobin; 3, carbonic anhydrase; 4, ovalbumin; 5, albumin bovine serum.

Figure 4A shows representative time courses of tryptic digestions of TgCEN1-C and TgCEN2-C in the absence and presence of Ca^2+^. In the case of apo-TgCEN1-C, the band representing the intact protein disappeared within 60-min and lower molecular mass bands were evident after a 1-min reaction. Upon binding of Ca^2+^ ions, TgCEN1-C underwent a change of susceptibility to trypsin, becoming more resistant than its apo-form to proteolytic digestion, since the band corresponding to the intact protein was evident following a 2-h reaction. On the other hand, parallel experiments performed with TgCEN2-C showed that in the presence or absence of Ca^2+^, the banding patterns are quite similar, indicating that trypsin has a similar access to the apo- and holo-conformation of the protein. It is worthwhile underlining that the apo-proteins are affected by proteolysis in a different way. Apo-TgCEN1-C is more easily digested by trypsin and is entirely hydrolyzed to lower molecular mass peptides, while apo-TgCEN2-C is almost completely resistant to proteolytic attack, as illustrated by the presence of the intact protein even after a 2-h reaction. Collective data from digestion correlate well with the results obtained from NMR and CD spectroscopy, providing evidence that significant differences exist within the structural conformations of the two centrin isoforms. Apo-TgCEN1-C is likely more flexible and less stable than apo-TgCEN2-C and Ca^2+^ binding has a significant effect on the conformation of TgCEN1-C, while it has only a subtle impact on the conformational properties of TgCEN2-C.

We next performed SEC of TgCEN1-C and TgCEN2-C in the presence of EGTA or Ca^2+^. As shown in Figure 4B, both apo-TgCEN1-C and apo-TgCEN2-C appeared as a single peak indicative of apparent molecular masses of 30.1 and 26.6 kDa respectively. The apo-forms of TgCEN1-C and TgCEN2-C revealed an apparent molecular mass that was ∼1.6-fold higher than their calculated molecular mass. However, it is well known that SEC separation for Ca^2+^-sensor proteins is largely based on alteration in their solvation properties and not just on their molecular weight. Indeed, many Ca^2+^ sensors, such as CaM and CaM-like proteins from different organisms [27], have atypical migrations in SEC, resulting in a characteristic overestimation of the molecular weight due to their elongated shape, when globular shape standards are used for column calibration. Thus, the higher apparent molecular mass observed in apo-centrins suggests that they likely possess extended conformations. Moreover, this data pointed to a monomeric form for both proteins, consistent with the presence of a single band on native PAGE.

Considering the retention time, Ca^2+^ is likely to induce conformational variations of TgCEN1-C toward a slightly more elongated shape, while no major effects are observed in the hydrated shape of TgCEN2-C. 

### 3.5. Interaction of TgCEN1-C and TgCEN2-C with ANS

**Figure 5:**
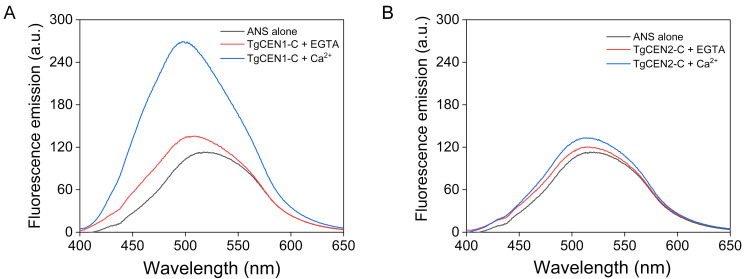
Fluorescence emission of ANS. Representative ANS fluorescence spectra of TgCEN1-C (**A**) and TgCEN2-C (**B**) in the presence of 2 mM EGTA (red) or 2 mM CaCl_2_ (blue). The spectra of ANS alone (black) is also shown.

ANS-based fluorescence was used to measure the degree of exposed hydrophobic patches in Ca^2+^ free and Ca^2+^ loaded centrins. When ANS interacts with hydrophobic regions on proteins, it displays a blue shifted emission and an increase in emission intensity. As shown in Figure 5A, considering the emission of ANS alone as a reference, when Ca^2+^ binds to TgCEN1-C, a large blue-shift (24 ± 3 nm) in the maximum emission wavelength of the probe associated with an evident increase in maximum intensity (2.4 ± 0.3) was observed, likely due to a marked increase in hydrophobic exposure. Notably, there were some modifications in ANS fluorescence even in the presence of EGTA, suggesting that apo-TgCEN1-C also exhibits hydrophobic pockets, which are, however, smaller than those in the holo-protein. On the other hand, only a small blue-shift in emission wavelength (6 ± 1 nm) and a non-significant increase in ANS fluorescence (1.2 ± 0.1) were observed for TgCEN2-C in the presence of Ca^2+^ (Figure 5B) compared to the spectrum of ANS alone. Thus, Ca^2+^ likely does not significantly change the amount of exposed hydrophobic regions in TgCEN2-C.

Binding of Mg^2+^ led to emission spectra for both centrins that were virtually identical to those in the presence of EGTA, therefore indicating the absence of specific effects of Mg^2+^ on exposure of hydrophobic patches (Figure S5).

### 3.6. Energetics of Ca^2+^ Binding 

**Figure 6:**
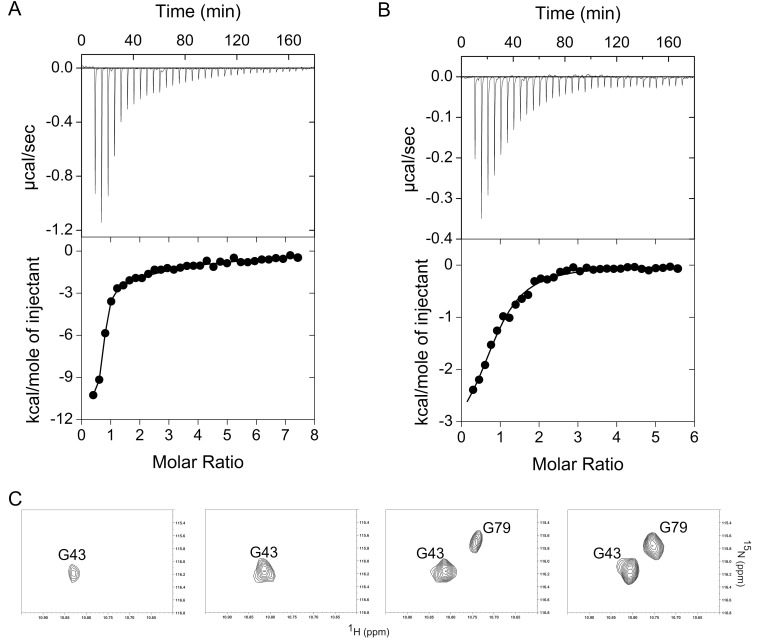
Ca^2+^ binding to TgCEN1-C and TgCEN2-C. (**A**,**B**) Representative Ca^2+^ titration of apo-TgCEN1-C (**A**) and apo-TgCEN2-C (**B**) as monitored by ITC. Representative raw heat-power changes (upper panels) and integrated binding isotherms (bottom panels). A first injection of 0.5 μL was made and then the first data point was removed from data fitting. Curve fitting was performed by considering the two binding sites model for TgCEN1-C and the one site model for TgCEN2-C. The protein concentration was 70 μM and 100 μM, for apo-TgCEN1-C and apo-TgCEN2-C, respectively. (**C**) The downfield region of the ^1^H-^15^N HSQC NMR spectra of TgCEN1-C recorded as a function of increasing Ca^2+^ concentration. The molar ratio of Ca^2+^:protein in each case was 0.9, 1.5, 4, and 10 (from left to right).

We attempted to measure the affinity for Ca^2+^ of both TgCEN1-C and TgCEN2-C using isothermal titration calorimetry (ITC). Representative isotherms for Ca^2+^ binding to isolated TgCEN1-C and TgCEN2-C are shown in Figure 6A,B, respectively, and optimal thermodynamic parameters are presented in Table 1.

Both thermograms of TgCEN1-C and TgCEN2-C for Ca^2+^ binding showed exothermic profiles. The TgCEN1-C data best fit a model that predicted two sets of sites for Ca^2+^ binding due to a two phases curve: one high-affinity site (*K*_d1_ of ~2.1 µM) and a second site with lower affinity (*K*_d2_ of ~26 µM). On the other hand, the Ca^2+^-binding isotherm for TgCEN2-C exhibited a classical sigmodal shape and the best fit was obtained using a simple one site model, which results in the binding of a single Ca^2+^ ion (binding stoichiometry, *n*  =  0.8 ± 0.1) with low affinity (*K*_d_ of ~63 μM). These ITC results are consistent with NMR experiments, supporting the binding of Ca^2+^ ions to EF-1 and EF-2 sites in TgCEN1-C and to EF-1 in TgCEN2-C.

Of note, the presence of two Ca^2+^-binding sites with different affinity in TgCEN1-C was clearly supported by NMR spectra. Ca^2+^ was titrated into apo-TgCEN1-C and ^1^H-^15^N HSQC spectra were recorded at each step. As visible in Figure 6C, the first signal to appear was that of the Ca^2+^-bound G43 (EF-1), while the peak of G79 (EF-2) appeared only at higher Ca^2+^:protein ratios. These observations prompted us to assign the dissociation constants determined from ITC *K*_d1_ to EF-1 (higher affinity Ca^2+^-binding site), and *K*_d2_ to EF-2 (lower affinity Ca^2+^-binding site).

### 3.7. Turbidity Measurements

**Figure 7:**
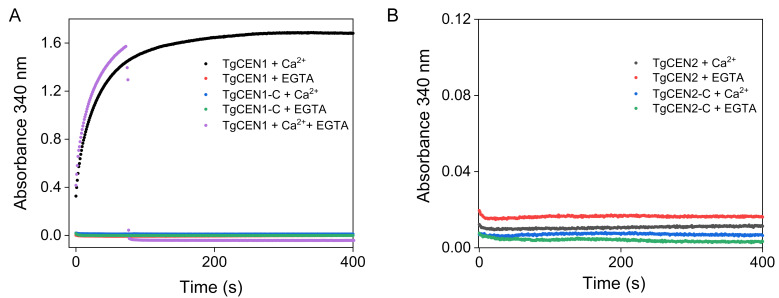
Turbidity measurements of full-length and truncated TgCEN1 (**A**) and full-length and truncated TgCEN2 (**B**). Scattering intensity at 340 nm was measured as a function of time at 37 °C for the samples (20 μM protein) with 1 mM Ca^2+^ or 1 mM EGTA. (**A**) Representative association profiles of Ca^2+^-bound full-length TgCEN1 (black), apo-full-length TgCEN1 (red), Ca^2+^-bound TgCEN1-C (blue) and apo-TgCEN1-C (green). The effect of EGTA on a solution of Ca^2+^-saturated full-length TgCEN1 is also shown (light violet). (**B**) Representative association profiles of Ca^2+^-bound full-length TgCEN2 (black), apo-full-length TgCEN2 (red), Ca^2+^-bound TgCEN2-C (blue) and apo-TgCEN2-C (green).

In order to investigate whether toxoplasma centrins have the tendency to self-assemble as a consequence of Ca^2+^ addition and the role of the N-terminal 20–25 residue extension in mediating this process, phenomena already described for many other centrins [36,49,50], turbidity measurements were performed by monitoring the absorbance at 340 nm in 20 mM Tris–HCl, 20 mM KCl pH 7.5 (and 0.5 mM DTT for TgCEN1) at 37 °C. All four toxoplasma protein variants available in our laboratory were tested, i.e., full-length TgCEN1, full-length TgCEN2, TgCEN1-C (the truncated form of TgCEN1 lacking the first 21 residues), and TgCEN2-C (the truncated form of TgCEN2 lacking the first 22 residues). As shown in Figure 7, in the absence of Ca^2+^, no changes in light scattering were observed for any of the variants. However, upon addition of 1 mM Ca^2+^, full-length TgCEN1 exhibited a considerable increase in turbidity (Figure 7A, black), indicating a higher propensity for this protein to associate into high-density material. Under the same experimental conditions, the presence of Ca^2+^ did not alter light scattering for full-length TgCEN2 (Figure 7B, black), truncated TgCEN1-C (Figure 7A, blue), and truncated TgCEN2-C (Figure 7B, blue). The observed association process with full-length TgCEN1 is Ca^2+^ dependent and reversible. Upon addition of EGTA to Ca^2+^-saturated full-length TgCEN1, the sample was again optically transparent, and the absorbance at 340 nm was reduced significantly (Figure 7A, light violet), indicating the disappearance of scattering particles. Importantly, the truncated variant TgCEN1-C showed no turbidity changes upon addition of Ca^2+^ (Figure 7A, blue), implying a key role of the N-terminal 21-residues extension in the Ca^2+-^dependent supramolecular assembly of full-length TgCEN1.

## 4. Discussion

Ca^2+^ ions act as second messengers for numerous signaling pathways in eukaryotic cells. Ca^2+^ signaling has been demonstrated to initiate the major events, invasion, motility, and egress of the lytic cycle in *T. gondii* [51]. However, the mechanisms by which this parasite regulates Ca^2+^ fluctuations at a molecular level to induce highly specific responses have not been thoroughly investigated. The presence in *T. gondii* of approximately 68 distinct genes predicted to encode proteins possessing at least one EF-hand motif suggests the large contribution of Ca^2+^-binding proteins in controlling intracellular free Ca^2+^ concentrations and regulating biological responses relevant for the infection cycle of the parasite [52]. Most of these proteins have not been characterized; however, the comprehension of their roles, and especially Ca^2+^ dependence, requires an exhaustive characterization of the molecular features of the proteins and of their interactions with this ion. 

Herein, we report on the characterization of two EF-hand motif-containing proteins from *T. gondii*, TgCEN1 and TgCEN2 centrins. TgCEN1 and TgCEN2 were produced in a recombinant form to study their Ca^2+^ binding characteristics as well as the effect of Ca^2+^ on protein conformation. Our studies revealed a different Ca^2+^ relay mode for each protein, reflecting the presence in toxoplasma of a Ca^2+^ signaling system with different biochemical properties. 

Toxoplasma centrins are associated with different cytoskeletal structures [19], but their possible Ca^2+^ regulation has never been established. Our NMR data, based on the use of Gly-6 as indicators of Ca^2+^-binding sites, coupled with ITC results and complemented with sequence analysis data, revealed that TgCEN1 binds two Ca^2+^ ions through its N-terminal domain composed of EF-1 and EF-2 with high/medium affinity (*K*_d1_ of ~2.1 µM and *K*_d2_ of ~26 µM, respectively), while TgCEN2 binds only one Ca^2+^ ion through EF-1 with low affinity (*K*_d_ of ~63 µM). Thus, both isoforms likely have only a subset of sites that are active. In both cases Mg^2+^ has no effect on these Ca^2+^ sites, which can be considered Ca^2+^ specific. Interestingly, centrins display large variance in their Ca^2+^ sensing abilities. *Chlamydomonas reinhardtii* centrin possesses two N-terminal high affinity Ca^2+^ binding sites and one site with lower affinity in the C-terminus [40]. Yeast centrin has three functional EF-hands, one N-terminal high affinity site and two C-terminal low-affinity sites [4]. HsCEN3 binds one Ca^2+^ with high affinity in the N-terminal lobe and two Ca^2+^ ions with low affinity in the C-terminal domain [13]. HsCEN2 has only one functional Ca^2+^ binding site located in EF-4 [53]. *Arabidopsis thaliana* CEN2 binds four Ca^2+^ ions, two with high affinity at EF-1 and EF-2 and two with lower affinity at EF-3 and EF-4 [6]. Centrin 4 from *Trypanosoma brucei* binds to Ca^2+^ with high affinity through its EF-3 and EF-4 in the C-terminal domain [15]. Thus, there is poor evolutionary conservation of Ca^2+^ sites among centrin proteins. It was noteworthy that EF-4 in both TgCEN1 and TgCEN2 was non-functional in terms of Ca^2^^+^ binding, even if it does conform to the EF hand consensus sequence. This finding is reminiscent of the diversities between predicted and experimentally measured binding sites observed for other Ca^2+^ binding proteins [25,41,54,55]. Of note, the presence of an aspartic acid residue instead of a glutamic acid residue at position 12 of EF-4 in TgCEN2, which is reported to greatly decrease the Ca^2+^-binding affinity and selectivity in many Ca^2+^ binding proteins [16,17,33], could be the reason why, in TgCEN2, only EF-1 was found to be a functional Ca^2+^ binding site. However, we cannot exclude the possibility that the Ca^2+^-binding characteristics (affinity and number of active binding sites) of TgCEN1 and TgCEN2 are likely undervalued in our in vitro conditions and other factors such as the presence of putative interactors can significantly affect the in vivo Ca^2+^ sensing abilities of the two isoforms. For instance, it has been suggested that in the case of the Ca^2+^-dependent HsCEN2–melittin interaction, a non-functional EF-hand in the N-terminal domain of the protein may be activated to bind Ca^2+^ when a complex is formed [53].

The results of the present work clearly demonstrate that TgCEN1 displays key properties of Ca^2+^ sensors in terms of Ca^2+^-induced conformational changes, including, upon the addition of Ca^2+^: (i) a significant increase in the elliptic signal likely due to changes in the interhelical angles within the EF-hand domains; (ii) considerable changes in the ^1^H-^15^N HSQC NMR spectra with the protein that adopts a more stable tertiary conformation; and iii) exposure of hydrophobic patches as indicated by ANS probe. It should be noted that for proteins that possess self-assembly properties, Ca^2+^-induced changes in ANS may not be due to conformational change, but rather could be due to binding between molecules in the assembly process. However, here we reported ANS experiments only for the truncated versions of toxoplasma centrins (TgCEN1-C and TgCEN2-C), which showed no detectable turbidity changes, and thus no tendency to self-assemble upon addition of Ca^2+^ (Figure 7). Importantly, all ANS experiments were conducted in a high ionic strength buffer (50 mM Tris-HCl, 150 mM KCl pH 7.5) at a protein concentration of 1 μΜ, whereas turbidity measurements were performed in a low ionic strength buffer (20 mM Tris-HCl, 20 mM KCl pH 7.5) at a protein concentration of 20 μΜ. Under conditions of high ionic strength, no self-assembly was observed upon addition of Ca^2+^ even for full-length TgCEN1 (the only protein variant that exhibited a considerable increase in turbidity in our experiments) (Figure S6A). Moreover, dynamic light scattering (DLS) experiments, performed at the conditions of high ionic strength of ANS experiments, allowed us to exclude the presence of small aggregates that we cannot distinguish in the turbidity studies for the truncated variants TgCEN1-C and TgCEN2-C (Figure S6B). These observations indicate that the assembly process is dependent on ionic strength, in line with a previous study of HsCEN2 [36], and confirm that the observed changes in ANS are due to increased exposure of the hydrophobic surface. This hydrophobic exposure is a thermodynamically unfavorable process; therefore, it is likely that the hydrophobic patch of TgCEN1 may be essential for interaction with other proteins, which is crucial for the function of Ca^2+^ sensors. Moreover, the apparent Ca^2+^-affinity of TgCEN1 is consistent with Ca^2+^-sensitive regulation of the protein, since the dissociation constants were in the low micromolar range (resting cytosolic Ca^2+^ concentration in toxoplasma is ~50–100 nM [56], as for other eukaryotic cells).

Therefore, as a Ca^2+^ sensor, TgCEN1 is supposed to interact and regulate downstream proteins. Unfortunately, information about the possible biological roles of TgCEN1 is still missing. In this scenario, the Ca^2+^ dependent ability of TgCEN1 to self-assemble observed in this work may account for crucial biological functions of the protein. Ca^2+^-dependent self-assembly seems to be a peculiar property of centrins and many cell biology studies have shown that centrins are crucial structural components of Ca^2+^-sensitive contractile filament systems. TgCEN1 is predominately localized to the highly regular structure of centrioles [19], suggesting that it may also participate in supramolecular organization. Thus, in the analysis of the functional role or of any potential interacting partner of TgCEN1, the self-association properties of the protein should be taken into account. In particular, our data indicate that the 21 amino acid region at the N-terminus of TgCEN1 has a fundamental role in protein association. It is likely that, as for human HsCEN2 [36], both ionic forces (interaction between the positive charges of the N-terminal and negative charges of the C-terminal regions of different subunits) and hydrophobic forces play a crucial role in the process of self-assembly. However, future work is needed to unravel the driving force and molecular mechanism of TgCEN1 self-association. 

While TgCEN1 behaves biochemically as a Ca^2+^ sensor, binding of Ca^2+^ to TgCEN2 did not cause a large conformational change, which is a hallmark of Ca^2+^ sensors, as demonstrated by NMR, CD, and ANS analysis. The NMR spectral fingerprint of Ca^2+^-bound TgCEN2 is distinct from that of the apo-state, although the differences are more consistent with only localized conformational rearrangements, likely within the single functional Ca^2+^ site. The very small changes in secondary structure, as indicated by CD spectra, do not preclude per se that Ca^2+^ binding induces conformational variations. However, following Ca^2+^ binding, no hydrophobic surfaces are exposed in TgCEN2-C, as shown by ANS fluorescence. Thus, although Ca^2+^ binds to TgCEN2-C, it does not drive the conformational change with the exposure of a hydrophobic region that is crucial for hydrophobic interaction with target proteins. Moreover, ITC experiments indicated a single Ca^2+^ binding site with a dissociation constant of ~63 μM in TgCEN2-C. Functional EF-hands are usually paired (the basic functional unit is not the EF-hand motif but rather the paired EF-hand domain) and possess affinity in the low micromolar range [16]. However, an EF-hand Ca^2+^ sensor domain can function even if it has only one site capable of binding Ca^2+^. There are many examples of functional Ca^2+^ sensor domains that have only one EF-hand that is able to bind Ca^2+^, including some previously studied centrins [4,13,53,57]. Moreover, for a Ca^2+^ binding protein, it is fundamental to consider the difference between affinity for Ca^2+^ by itself versus its affinity for Ca^2+^ in the presence of physiological targets. Indeed, binding partners can have an enormous effect on measurements of Ca^2+^ affinities since the two binding events are energetically coupled. Consequently, vast increases in Ca^2+^ affinities were often observed when the measurements are made in the presence of a partner protein or even just the interaction motif peptide, as in the case of yeast, plant and green algal centrins [7,14,58]. Thus, a site that appears to have low Ca^2+^ affinity when measured for the isolated protein, such as for TgCEN2, may well be fully functional. Moreover, it is possible that the Ca^2+^ binding properties of TgCEN2 can be tuned differentially by diverse target proteins.

The absence of increased exposure of hydrophobic regions in TgCEN2 could also imply it has a role as a Ca^2+^ buffering protein. In fact, exposure of hydrophobic surfaces would be unfavorable for these proteins because it may limit their stability and Ca^2+^ affinity. However, the low affinity for Ca^2+^ suggests that TgCEN2 is unlikely to work as a Ca^2+^-buffer protein, since Ca^2+^-buffering proteins usually have a very high affinity for Ca^2+^ to efficiently chelate the ion (e.g., parvalbumin with K_d_ ~50 nM [59]).

Thus, the mechanism of action of TgCEN2 is not clear since it behaves neither as a Ca^2+^ buffering protein nor as a typical Ca^2+^ sensor protein. It is not known how large the impact of Ca^2+^ on protein structure has to be in order to be physiologically relevant and we cannot exclude the possibility that a different Ca^2+^-relay mode and different ways of interaction with target proteins exist that do not follow the paradigm of a Ca^2+^-dependent switch with exposure of hydrophobic surfaces. Notably, as demonstrated by limited proteolysis and CD denaturation experiments, TgCEN2 is very stable even in the apo-form, suggesting the possibility that apo-TgCEN2 might also bind to a potential target and that this interaction may play a significant role in the Ca^2+^ signaling pathway. 

Moreover, TgCEN2 does not display the tendency to self-assemble, underlining the molecular differences with TgCEN1, and thus indirectly highlighting different roles in Ca^2+^ regulated processes. TgCEN2 may not be involved in the crucial cellular process requiring self-assembly of centrins. Accordingly, TgCEN2, in addition to centrioles, is also targeted to the apical (the preconoidal rings) and basal ends (the basal complex) of the parasite, in addition to annuli located at the lower part of the apical cap within the membrane cortex. This differential distribution of TgCEN2 points to versatile functions of TgCEN2 in the four distinct structures. Experimental evidence based on gradual depletion and knockdown of TgCEN2 revealed that this protein is involved in several Ca^2+^-mediated processes, namely microneme secretion, motility, host cell attachment, invasion, and egress [21]. In addition, these analyses also indicate that the depletion of TgCEN2 from the four locations follows different kinetics, first from the pre-conoidal rings and peripheral annuli, leading to major invasion defects, and later from basal complex and centrioles, resulting in replication deficiency [21,22]. In this scenario, identifying the physical interactors of TgCEN2 and characterizing the magnitude of the reciprocal interactions between Ca^2+^, TgCEN2 and TgCEN2-binding targets will be crucial to unravel the exact Ca^2+^-sensing properties, mode of action and biological function of this centrin isoform.

## 5. Conclusions

In summary, our results suggest the existence of a sophisticated interplay between Ca^2+^ and TgCEN1 and TgCEN2, with each centrin isoform displaying a precise detection mode for Ca^2+^. Toxoplasma occupies a unique phylogenetic position since it is an early branching eukaryote, and therefore, the characterization of the molecular properties, mechanism of action, and functional specialization of parasitic centrins and, more in general of Ca^2+^ binding proteins, can provide useful information about the early evolution of Ca^2+^ signaling machinery. Moreover, it may be relevant in the discovery of promising druggable targets to treat toxoplasmosis.

## Figures and Tables

**Table 1:** Thermodynamic parameters for Ca^2+^ binding to TgCEN1-C and TgCEN2-C. The mean values ± SEM from triplicate experiments using at least two different protein preparations are presented.

	Binding Sites Used for Modelling	n	K_a_ (M^−1^)	ΔH (kcal mol^−1^)
TgCEN1-C	2	n_1_ = 0.7 ± 0.1	4.8 × 10^5^ ± 6.1 × 10^3^	−9.6 ± 1.1
		n_2_ = 1.2 ± 0.3	3.9 × 10^4^ ± 4.5 × 10^3^	−3.2 ± 0.7
TgCEN2-C	1	0.8 ± 0.1	1.6 × 10^4^ ± 1.5 × 10^3^	−3.4 ± 0.4

## Supplementary Materials

The following are available online at https://www.mdpi.com/2218-273X/10/8/1142/s1, Table S1: List of mutagenic primers used to generate TgCEN1 and TgCEN2 variants, Figure S1: SDS- and native-PAGE analysis of truncated (A, B) and full-length (C, D) variants of toxoplasma centrins.; Figure S2: ^1^H-^15^N HSQC NMR spectra of full-length TgCEN2; Figure S3: ^1^H-^15^N HSQC NMR spectra of TgCEN1-C and TgCEN2-C in the presence of MgCl2, Figure S4: Far-UV CD spectra of TgCEN1-C and TgCEN2-C in the presence of MgCl_2_, Figure S5: Fluorescence emission of ANS in the presence of MgCl_2_, Figure S6: Dependence on ionic strength of self-assembly process.
