# Supplementary material for: Distinct Calcium Binding and Structural Properties of Two Centrin Isoforms from Toxoplasma gondii
Source: Biomolecules. 2020 Aug 4;10(8):1142. doi: 10.3390/biom10081142 (PMC7465447; doi:10.3390/biom10081142)
Supplement: Supplementary file 1 [file biomolecules-10-01142-s001.zip › biomolecules-857725-supplementary.pdf]

Supplementary Material

Distinct Calcium Binding and Structural Properties of Two Centrin Isoforms from *Toxoplasma gondii*

Luca Bombardi†, Marco Pedretti†, Carolina Conter, Paola Dominici and Alessandra Astegno*

Department of Biotechnology, University of Verona, Strada Le Grazie 15, 37134 Verona, Italy.

†These authors contributed equally to the work.
*Corresponding author: Alessandra Astegno, e-mail: alessandra.astegno@univr.it TEL +39 0458027955

Supplementary Table S1. List of mutagenic primers used to generate TgCEN1 and TgCEN2 variants. PF = Primer Forward, PR = Primer Reverse.

Mutation	Type of Primer	Primer Sequence
**TgCEN1-C**	PF	CCACCACCACCACCACTTGACGGAAGAACAGC
	PR	GGTGGTGGTGGTGGTGAAGTGCCTTCTTGTCG
**TgCEN2-C**	PF	CCACCACCACCACCACCTAACAGAGGACGAAATC
	PR	GGTGGTGGTGGTGGTGGATTGTCTCCTGCTTTAG
**G43A TgCEN1-C**	PF	CACAGATGGGTCAGCCTGCATCGACGCAA
	PR	TTGCGTCGATGCAGGCTGACCCATCTGTG
**G79A TgCEN1-C**	PF	CAAGGATGGCACGGCAAGCGTTGACTTCC
	PR	GGAAGTCAACGCTTGCCGTGCCATCCTTG
**G152A TgCEN1-C**	PF	CGGGACGGCGACGCGGAAATCAATGAAG
	PR	CTTCATTGATTTCCGCGTCGCCGTCCCG
**G44A TgCEN2-C**	PF	GACACAGACGGCAGTGCTATGATCGATCCGAAA
	PR	TTTCGGATCGATCATAGCACTGCCGTCTGTGTC
**G153A TgCEN2-C**	PF	GACTCAAATGGCGACGCGGAGATTTCCTTCGAG
	PR	CTCGAAGGAAATCTCCGCGTCGCCATTTGAGTC

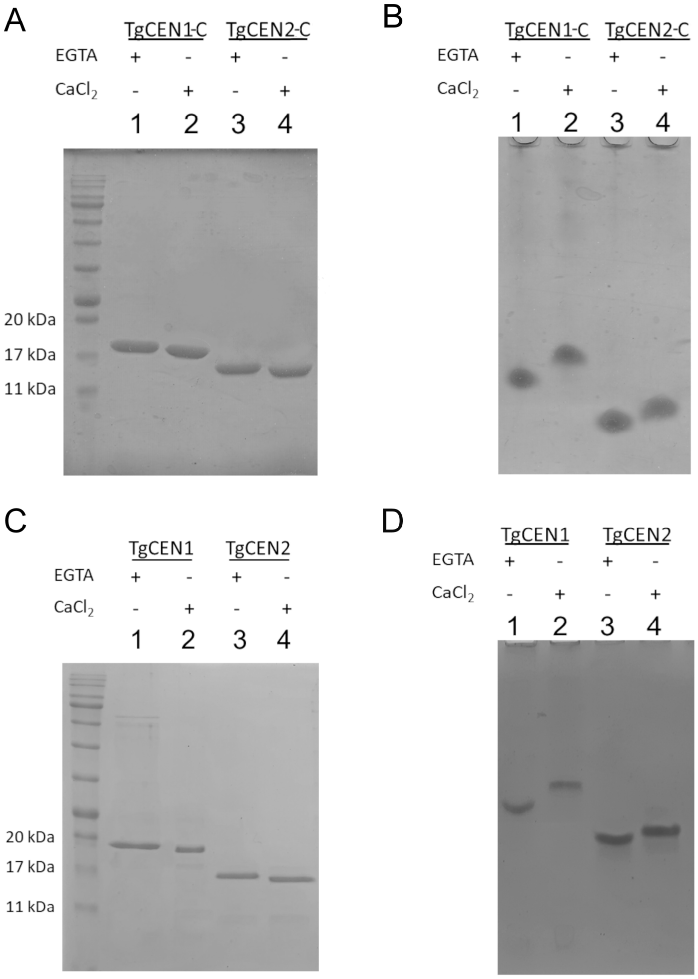

Figure S1. **SDS- and native-PAGE analysis of truncated (A, B) and full-length (C, D) variants of toxoplasma centrins.** The purified proteins were analyzed on 15% SDS-PAGE **(A, C)** and 12% native-PAGE **(B, D)** in the presence of 2 mM EGTA or 2 mM $CaCl_2$.

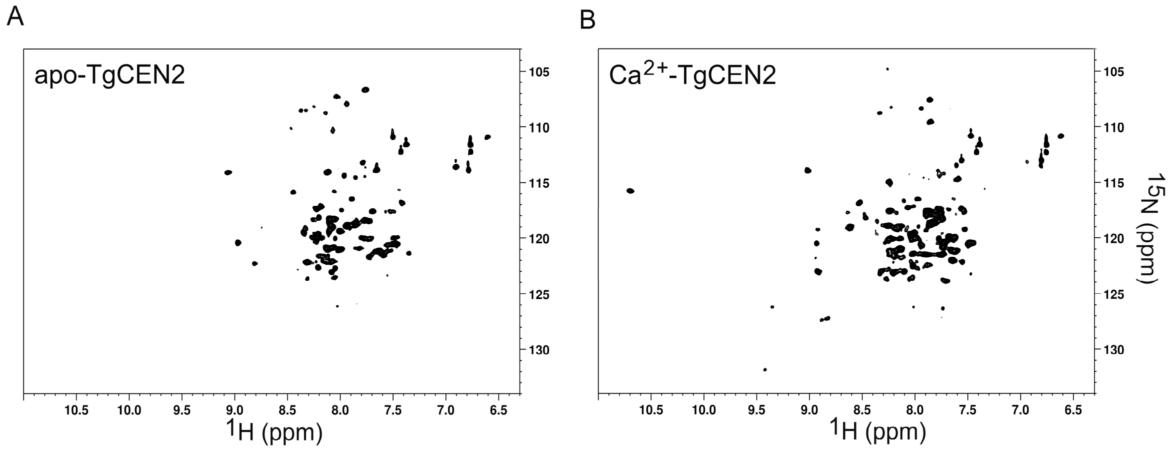

Figure S2. **^{1}H-^{15}N HSQC NMR spectra of full-length TgCEN2.** The apo-condition was obtained by adding 5 mM EGTA (A), while the Ca^{2+}-bound condition was obtained by adding 5 mM $CaCl_2$ (B).

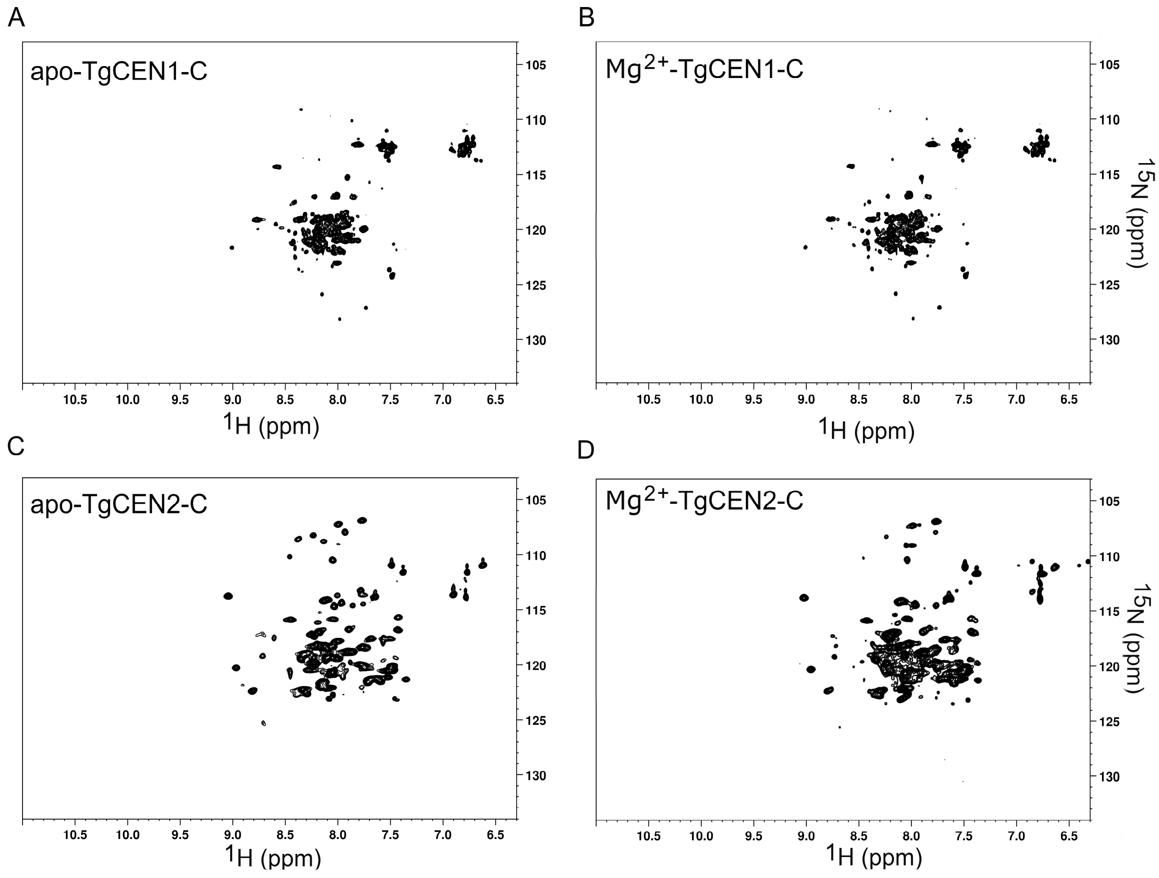

Figure S3. ^{1}H-^{15}N HSQC NMR spectra of TgCEN1-C and TgCEN2-C in the presence of $MgCl_2$. The apo-condition was obtained by adding 5 mM EGTA, while the Mg^{2+}-bound condition was obtained by adding 5 mM $MgCl_2$.

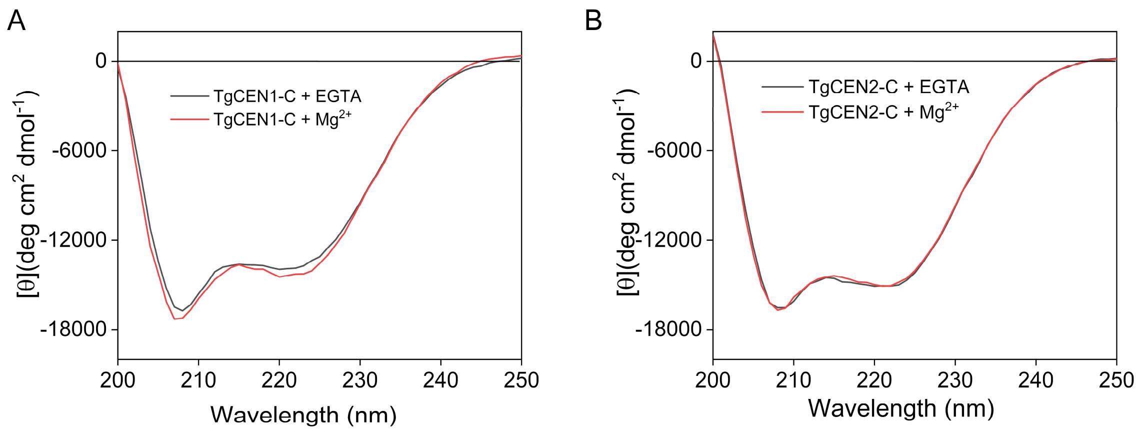

Figure S4. Far-UV CD spectra of TgCEN1-C and TgCEN2-C in the presence of $MgCl_2$. Far-UV CD spectra of 0.2 mg/mL TgCEN1-C (**A**) and TgCEN2-C (**B**) in the presence of 2 mM EGTA (black) and 0.5 mM EGTA+ 2 mM $MgCl_2$ (red).

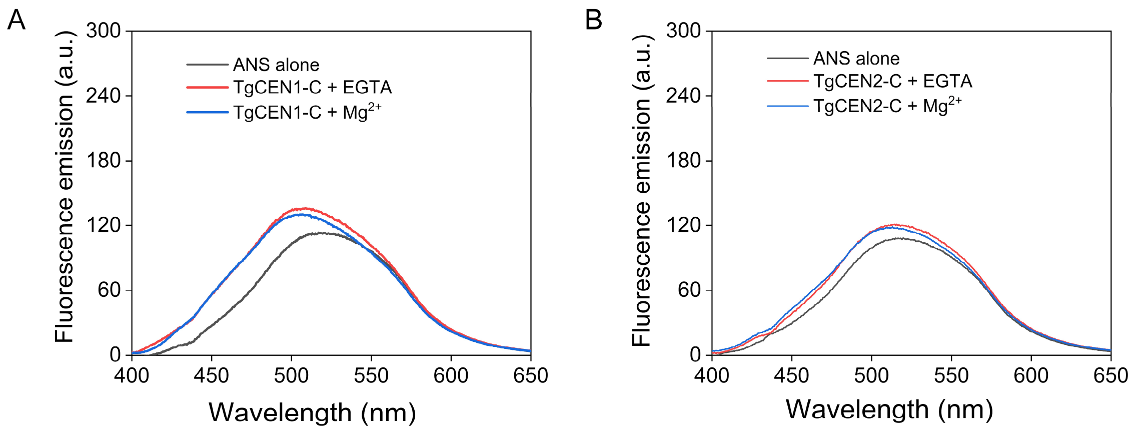

Figure S5. Fluorescence emission of ANS in the presence of $MgCl_2$. Representative ANS fluorescence spectra of TgCEN1-C (**A**) and TgCEN2-C (**B**) in the presence of 2 mM EGTA (red) or 0.5 mM EGTA+ 2 mM $MgCl_2$ (blue). The spectra of ANS alone (black) is also shown.

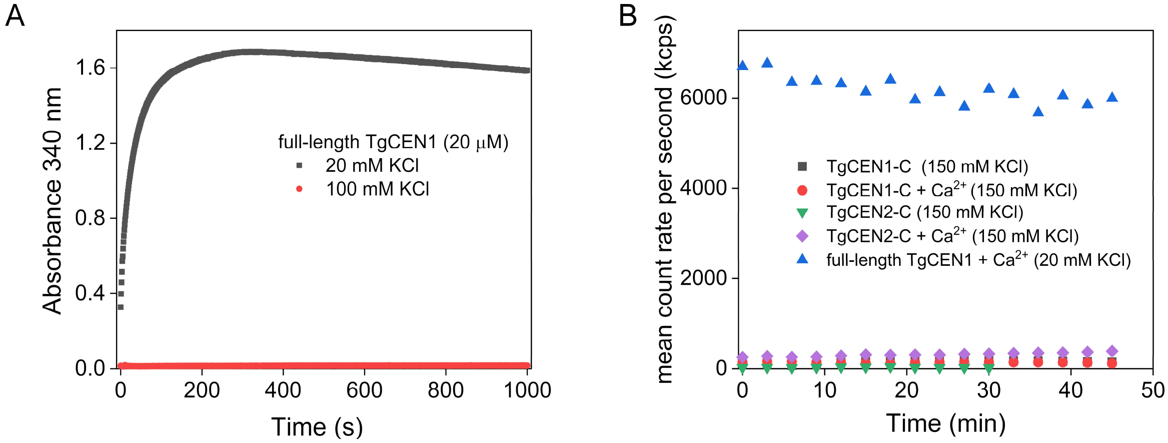

Figure S6. Dependence on ionic strength of self-assembly process. (**A**) Turbidity measurements of full-length TgCEN1 in 20 mM Tris–HCl pH 7.5 at low and high ionic strength. Scattering intensity at 340 nm was measured as a function of time at 37°C for the samples (20 μM protein) upon addition of 1 mM Ca^{2+}. A dependence on ionic strength of the assembly process of full-length TgCEN1 was clearly observed. **(B)** Time-dependent changes of the total count rate (measured as kilocounts per second) of deleted variants TgCEN1-C and TgCEN2-C before and after addition of Ca^{2+} at high ionic strength (150 mM KCl) as used in ANS experiments. No self-assembly was observed. For comparison, the profile of full-length TgCEN1 that shows high aggregation propensity upon Ca^{2+} addition at low ionic strength (20 mM KCl) was reported.
